# COVID-19 in Older Individuals Requiring Hospitalization

**DOI:** 10.3390/idr14050074

**Published:** 2022-09-12

**Authors:** Petros Ioannou, Despoina Spentzouri, Myrto Konidaki, Michalis Papapanagiotou, Sotiris Tzalis, Ioannis Akoumianakis, Theodosios D. Filippatos, Symeon Panagiotakis, Diamantis P. Kofteridis

**Affiliations:** COVID-19 Department, University Hospital of Heraklion, 71500 Heraklion, Greece

**Keywords:** COVID-19, SARS-CoV-2, older, elderly, geriatric

## Abstract

Older individuals have an increased risk for severe coronavirus disease 2019 (COVID-19) and a higher risk for complications and death. The aim of this study was to investigate the clinical characteristics of older patients admitted with COVID-19 and describe their outcomes. This was a retrospective cohort study of patients older than 65 years admitted to the COVID-19 Department of the University Hospital of Heraklion. Data recorded and evaluated included age, gender, Infectious Diseases Society of America (IDSA) severity score, Charlson comorbidity index (CCI), high-flow nasal oxygen (HFNO) use, admission to the Intensive Care Unit (ICU), laboratory exams, treatment administered, and outcome. In total, 224 patients were evaluated in the present study. The median age was 75 years and 105 (46.9%) were female. In 50 patients (22.7%), HFNO was used and 23 (10.3%) were admitted to the ICU. Mortality was 13.4% (30 patients). Patients that died had higher age, were more likely to be male, had an IDSA severity score of 3, had prior HFNO use, had been admitted to the ICU, and were also more likely to have a higher white blood cell (WBC) count, CRP, ferritin, procalcitonin, d-dimers, and troponin. A multivariate logistic regression analysis identified age and the need for HFNO use to be independently positively associated with mortality. To conclude, COVID-19 carries significant mortality in hospitalized older patients, which increases with age, while the need for HFNO also increased the likelihood of worse outcomes. Clinicians caring for patients with COVID-19 should bear in mind these two factors. Future studies could elaborate on the effect of new variants on the dynamics of mortality in older patients.

## 1. Introduction

Severe acute respiratory syndrome coronavirus 2 (SARS-CoV-2) has infected more than 570,000,000 people worldwide and has caused more than 6,400,000 deaths [1]. However, not all people carry the same risk for severe disease and fatal outcomes. For example, patients with chronic comorbidities or conditions such as chronic kidney disease, chronic obstructive pulmonary disease, diabetes, hypertension, cancer, active smoking, obesity, older age, patients developing medical complications such as acute kidney injury, and patients with other parameters such as increased d-dimers or male gender often have with worse outcomes [2]. More specifically, older individuals have an increased risk for severe disease and are at higher risk for complications and death [3]. Likely contributing factors include social issues such as the fact that older individuals are far more likely to reside in nursing homes, leading to an increased likelihood of viral transmission as well as factors possibly associated with a higher potential for pathogenicity of the virus in older people such as the relative immunosenescence that comes with aging, due to a disruption in both innate and adaptive immunity, inflammaging, the inadequate clearance of viral particles by the ciliary function of the respiratory tract, and even factors associated with testosterone levels in older men [4,5,6]. For example, testosterone could exert a suppressive role in the functions of the immune system by acting on the androgen receptors of the immune cells, thus regulating gene expression. Activation of these receptors leads to a reduction in immune cell activity through the reduction in the expression of inflammatory mediators and the promotion of the expression of anti-inflammatory mediators by macrophages and T-cells [7,8]. Indeed, the age-related reduction in testosterone levels has been linked to a higher likelihood of infection-related hospitalization and mortality in patients with end-stage renal disease on hemodialysis [9]. On the other hand, downstream signaling of toll-like receptors (TLRs) may be impaired in older people, leading to inappropriate immune responses [10,11].

The aim of this study was to investigate the clinical characteristics of older patients admitted to a tertiary center with coronavirus disease 2019 (COVID-19) and to describe their outcomes.

## 2. Materials and Methods

### 2.1. Study Population

This study followed the retrospective cohort design, as previously published [12]. Study participants were older patients admitted to the COVID-19 Department of the University Hospital of Heraklion, Crete, Greece until August 2021. Patients were included if they were 65 years old or older, were diagnosed with COVID-19 with a positive reverse transcription-polymerase chain reaction (RT-PCR) test for severe acute respiratory syndrome coronavirus 2 (SARS-CoV-2), and were admitted to the COVID-19 department. Exclusion criteria were the absence of a positive RT-PCR test for SARS-CoV-2 and the transfer of patients to another hospital that did not allow for the completion of data collection. Data recorded and evaluated included age, gender, Infectious Diseases Society of America (IDSA) severity score, Charlson comorbidity index (CCI), high-flow nasal oxygen (HFNO) use, admission to the Intensive Care Unit (ICU), laboratory exams, treatment administered, and outcome.

The study was approved by the Ethics Committee of the University Hospital of Heraklion.

### 2.2. Statistics

Statistical analysis was performed depending on the type of data, as previously published [12,13]. More specifically, categorical data were analyzed with Fisher’s exact test. Continuous variables were compared using the Student’s t-test for normally distributed variables and the Mann–Whitney U-test for the non-normally distributed variables. All tests were two-tailed and *p* ≤ 0.05 were considered to be significant. Data are presented as the numbers (%) for categorical variables and medians [interquartile range (IQR)] or means [±standard deviation (SD)] for continuous variables. A linear regression analysis model was developed to evaluate the effect of several parameters (age, gender, IDSA severity score, CCI, high-flow nasal oxygen use, admission to the ICU, laboratory exams, and treatment administered) with mortality. All were calculated with GraphPad Prism 6.0 (GraphPad Software, Inc., San Diego, CA, USA). A multivariate logistic regression analysis model was developed to evaluate the association of factors identified in the univariate analysis with a *p* ≤ 0.05 with mortality. Multivariate analysis was performed using SPSS version 23.0 (IBM Corp., Armonk, NY, USA).

## 3. Results

In total, 224 patients older than 65 years old with COVID-19 admitted to the COVID-19 Department of the University Hospital of Heraklion were recorded and evaluated during the study period. Table 1 shows the characteristics of the included patients. The median age was 75 years and 105 (46.9%) were female. Median CCI was 4, and 86 (38.4%) of patients had an IDSA severity score of 3. HFNO use was deemed necessary and was used in 50 patients (22.7%), while 23 (10.3%) patients were admitted to the ICU. Table 1 also shows a comparison of the characteristics between male and female patients. More specifically, male older patients with COVID-19 had similar age, rate of IDSA score of 3, need for HFNO use, white blood cell count, procalcitonin, d-dimers, and troponin levels as well as the same rate of remdesivir or convalescent plasma use to female patients. However, male patients had significantly higher CCI, C-reactive protein (CRP), and ferritin levels and also had higher mortality.

Mortality was 13.4% (30 patients). Patients that died had higher age, were more likely to be male, had an IDSA severity score of 3, were more likely to have necessitated HFNO use or admission to the ICU, and had higher white blood cell (WBC) count, CRP, ferritin, procalcitonin, d-dimers, and troponin levels. Table 2 shows a comparison of the characteristics between the patients that died and patients that survived.

A univariate regression analysis identified male gender, higher age, higher IDSA severity score, higher CCI, need for HFNO use, ICU admission, lower lymphocytes, and higher platelets, CRP, ferritin, and troponin to be associated with higher mortality. However, a multivariate logistic regression analysis identified only age and the need for HFNO use to be independently positively associated with mortality. The results of the regression analysis are shown in Table 3.

## 4. Discussion

The present study retrospectively described a cohort of older patients who were hospitalized due to COVID-19 in a University Hospital. Patient characteristics were compared between male and female older patients and patients who survived with those who died, while parameters associated with mortality were evaluated through multivariate logistic regression analysis. Older age and the need for HFNO use were factors independently associated with higher mortality.

Older individuals are at an increased risk for complications and death due to COVID-19 [3,14,15,16,17]. This increased risk may be associated with several changes that come with age such as decreased mucociliary clearance of the respiratory tract, hormonal changes such as a reduction in the testosterone levels in older men, and immunological changes that may affect the patient’s ability to clear the virus [4,5,6,10,11]. For example, low testosterone levels that can be seen in older men have been associated with immunological changes that can lead to an increased likelihood of infection [9]. More specifically, low testosterone has been associated with a higher likelihood of severe COVID-19 pneumonia [18,19,20,21]. Additionally, mucociliary clearance in the respiratory tract is reduced in older individuals, as has been shown in a study among healthy volunteers of different ages in whom mucociliary clearance and ciliary beat frequency were calculated and were found to be lower in older individuals [5]. On the other hand, SARS-CoV-2 infection may lead to a decrease in the ACE2 protein levels, which may increase the expression of pro-inflammatory mediators, leading to increased severity and higher mortality of COVID-19 [22,23,24,25]. This is of particular importance in older patients, as ACE2 levels decline with age, making them more vulnerable to the immunologic consequences associated with the ACE2 level reduction induced by SARS-CoV-2 [26]. Moreover, anatomical changes in the lungs and the potential for atrophy of the respiratory muscles can be associated with changes in respiratory function, making older individuals more vulnerable to complications of COVID-19 due to a reduction in the respiratory reserve and decreased defense barriers. Furthermore, altered innate and adaptive immune responses due to aging such as decreased recognition of pathogens, chemotaxis, and phagocytosis as well as a reduced diversity of T-cell receptors that are responsible for the recognition of viral particles and reduced antibody secretion may also place older individuals at higher risk for complications by COVID-19 [27].

More than 30% of the confirmed COVID-19 cases in China have been above 60 years old, while in this age group, hypertension, diabetes, and cardiovascular disease are increasingly prevalent, with rates of 12.8%, 5.3%, and 4.2%, respectively [28]. In an analysis of patients from the USA, Europe, and Canada, patients with an age over 65 years accounted for more than 90% of people that succumbed to COVID-19, while 75% of people who succumbed were individuals older than 80 years old [23]. This is in agreement with the findings of the present study, where a multivariate logistic regression analysis identified that age was independently associated with mortality in patients with COVID-19 older than 65 years.

HFNO use is associated with a mortality benefit, a reduction in stay in the ICU, and a reduction in the duration of ventilation in patients with COVID-19 [29,30]. Herein, we found that the need for HFNO was identified as an independent factor associated with mortality in older patients with COVID-19. This implies that the development of severe hypoxemic respiratory failure is clearly associated with mortality in patients with COVID-19, as is clearly suggested by the literature [31,32,33,34,35]. For example, many of the studies published during the early stage after the onset of the COVID-19 pandemic have described respiratory failure as the leading cause of death [31,32,33]. The pathophysiology of severe hypoxemia in patients with COVID-19 typically involves the mechanism of acute respiratory distress syndrome (ARDS), which is associated with diffuse alveolar damage in the patients’ lungs [36]. However, dysregulation of the coagulation cascade in the context of the immune activation and formation of thrombi in the pulmonary vasculature has also been described [37].

Older patients with COVID-19 that died had worse laboratory findings compared to patients that survived in terms of lymphocyte number, ferritin, troponin, d-dimers, procalcitonin, CRP, and WBCs while some of these parameters have also been recognized by other studies [38,39,40]. For example, the d-dimer levels, CRP, and neutrophil-to-lymphocyte ratio have been recognized to be associated with a worse prognosis in patients with COVID-19 [39]. More specifically, on the admission of patients with COVID-19, a d-dimer level of 0.565 mg/L or higher was found to have a sensitivity and a specificity of 85.7% and 80.6% for worse outcomes, respectively. For CRP, a value of 0.0087g/L or higher had a sensitivity and a specificity of 81% and 62.5%, while a neutrophil to lymphocyte ratio of 3.21 or higher had a sensitivity and specificity of 84.4% and 62.8%, respectively, for worse outcomes in these patients. In a recent narrative review, several biomarkers that are associated with worse outcomes in COVID-19 were discussed [41]. For example, the association of increased troponin with worse cardiovascular outcomes, or the association of LDH and AST with worse pulmonary function, need for mechanical ventilation, and mortality have been described [41,42,43,44,45,46,47]. Increased troponin, either I or T, may reflect myocardial injury in patients with COVID-19 and has been shown to be associated with higher 30-day mortality when the measured levels were higher than the 99th percentile, the gender-specific 99th percentile [48]. Regarding serum LDH on admission, a value of 359.5 U/L had a sensitivity and a specificity of 93.8% and 88.2% for predicting death in COVID-19 patients, respectively [47].

Other factors associated with mortality such as frailty have also been described in other studies involving older patients with COVID-19. For example, in a recent systematic review including data from 924,520 patients, frailty was found to increase more than five-fold the likelihood of COVID-19-associated mortality [49]. These findings have been also verified by other studies [50,51].

Some of the limitations of this study were the relatively small sample size and the fact that the population of this study was derived from only one hospital, thus the generalization of the results may be limited.

## 5. Conclusions

This retrospective cohort study presented the clinical and laboratory characteristics of older patients hospitalized for COVID-19 in a single hospital. A comparison between patients who survived with those who succumbed revealed that patients who succumbed were older, more likely to be male, or have an IDSA severity score of 3, were more likely to have necessitated HFNO use or admission to the ICU, and had higher white blood cell (WBC) count, CRP, ferritin, procalcitonin, d-dimers, and troponin levels. Furthermore, a multivariate logistic regression analysis showed that older age and the need for HFNO use were factors independently associated with higher mortality in older patients with COVID-19. Clinicians should bear in mind these two factors when treating older individuals with COVID-19. Future studies could elaborate on the effect of new variants on the dynamics of mortality in older patients.

## Figures and Tables

**Table 1:** The characteristics of patients included in the study and the comparison of male and female patients.

	All Patients (*n* = 224)	Male (*n* = 119)	Female (*n* = 105)	*p*
Age, median (IQR) *	75 (70–81)	76 (70–81)	74 (69–81)	0.6248
Female, %	46.9			
CCI, median (IQR) **	4 (3–6)	5 (3–6)	4 (3–5)	0.0191
IDSA severity score 3, % ***	38.4	39.5	37.1	0.7835
HFNO, % ****	22.7	27.8	17.1	0.0761
ICU admission, % ^#^	10.3	13.6	6.7	0.1223
WBCs (/μL), median (IQR) ^##^	7200 (3575–12,550)	8400 (4225–13,400)	6150 (3375–11,750)	0.0650
CRP (mg/L), median (IQR) ^###^	7.4 (3.2–13.8)	8.5 (4.4–16.5)	6 (2.9–11.6)	0.0011
Ferritin (ng/mL), median (IQR)	621 (321.5–1252)	802 (376.8–1644)	421 (256–783)	<0.0001
Procalcitonin (ng/mL), median (IQR)	0.1 (0.05–0.27)	0.11 (0.05–0.32)	0.1 (0.04–0.22)	0.1396
D–dimers (ng/mL), median (IQR)	1.47 (0.77–3.89)	1.53 (0.76–4.22)	1.42 (0.77–3.31)	0.6721
Troponin (ng/mL), median (IQR)	10.3 (5.8–23.8)	11.3 (6.4–34.7)	9.6 (5.4–19.4)	0.0625
Remdesivir, %	65.6	63.1	68.3	0.4736
Convalescent plasma, %	12.1	13.5	10.7	0.5393
Mortality, %	13.4	18.5	7.6	0.0188

* IQR: interquartile range; ** CCI: Charlson comorbidity index; *** IDSA: Infectious Diseases Society of America; **** HFNO: high-flow nasal oxygen; ^#^ ICU: intensive care unit; ^##^ WBCs: white blood cells ^###^ CRP: c-reactive protein.

**Table 2:** The characteristics of the patients in terms of mortality.

	Survived (*n* = 194)	Died (*n* = 30)	*p*
Age, median (IQR) *	74 (69–79)	83.5 (76–90.3)	<0.0001
Female, %	50	26.7	0.0188
CCI, median (IQR) **	4 (3–6)	6 (5–7)	<0.0001
IDSA severity score 3, % ***	33	73.3	<0.0001
HFNO, % ****	18	57.7	<0.0001
ICU admission, % ^#^	8.2	24.1	0.0169
WBCs (/μL), median (IQR) ^##^	6600 (3575–11,925)	12450 (4425–16,775)	0.0306
CRP (mg/L), median (IQR) ^###^	6.7 (3–12)	14.9 (10–20.4)	<0.0001
Ferritin (ng/mL), median (IQR)	577 (309–1083)	1214 (615–2933)	0.0028
Procalcitonin (ng/mL), median (IQR)	0.09 (0.05–0.25)	0.27 (0.18–0.87)	0.0027
D–dimers (ng/mL), median (IQR)	1.35 (0.68–3.37)	3.67 (1.7–11.1)	0.0012
Troponin (ng/mL), median (IQR)	9.6 (5.4–18.3)	153.5 (38.1–693.8)	<0.0001
Remdesivir, %	66	62.5	0.8204
Convalescent plasma, %	10.5	25	0.0883

* IQR: interquartile range; ** CCI: Charlson comorbidity index; *** IDSA: Infectious Diseases Society of America; **** HFNO: high–flow nasal oxygen; ^#^ ICU: intensive care unit; ^##^ WBCs: white blood cells ^###^ CRP: c–reactive protein.

**Table 3:** The results of the logistic regression analysis of the mortality of the included patients.

	Univariate Analysis p	Multivariate Analysis p	OR ^##^ (95% CI) ^###^
Female gender	0.0171	0.501	0.466 (0.05–4.314)
Age (per year)	<0.0001	0.002	1.334 (1.108–1.606)
IDSA severity score *	0.0003	0.572	1.415 (0.424–4.717)
CCI (per unit) **	0.0001	0.740	1.081 (0.682–1.713)
HFNO use ***	0.0001	0.011	18.999 (1.944–185.644)
ICU admission ****	0.0085	0.283	0.094 (0.004–5.216)
Lymphocytes (per K/μL)	0.0073	0.117	0.138 (0.014–1.611)
Platelets (per K/μL)	0.0218	0.639	1 (1–1)
CRP (per mg/L) ^#^	0.0001	0.075	1.104 (0.99–1.236)
Ferritin (per ng/mL)	0.0002	0.076	1 (1–1.001)
Troponin (per ng/mL)	0.0001	0.059	1.001 (1–1.002)

* IDSA: Infectious Diseases Society of America; ** CCI: Charlson comorbidity index; *** HFNO: high-flow nasal oxygen; **** ICU: intensive care unit; ^#^ CRP: c-reactive protein; ^##^ OR: odds ratio; ^###^ CI: confidence intervals.

## Data Availability

The data presented in this study are available on request from the corresponding author.
